# JNK Signaling in *Drosophila* Aging and Longevity

**DOI:** 10.3390/ijms22179649

**Published:** 2021-09-06

**Authors:** Tian Gan, Lixia Fan, Long Zhao, Mala Misra, Min Liu, Min Zhang, Ying Su

**Affiliations:** 1 Institute of Evolution & Marine Biodiversity, Ocean University of China, Qingdao 266003, China; gantian@stu.ouc.edu.cn (T.G.); fanlixia@stu.ouc.edu.cn (L.F.); zhaolong@ouc.edu.cn (L.Z.); 2 College of Marine Life Sciences, Ocean University of China, Qingdao 266003, China; 3 College of Fisheries, Ocean University of China, Qingdao 266003, China; 4 Key Laboratory of Mariculture (OUC), Ministry of Education, Qingdao 266003, China; 5 Department of Biology, Washington College, Chestertown, MD 21620, USA; mmisra2@washcoll.edu; 6 Ministry of Education Key Laboratory of Cell Proliferation and Differentiation, School of Life Sciences, Peking University, Beijing 100871, China; liumin02@pku.edu.cn; 7 Division of Biomedical Sciences, University of California, Riverside, CA 92521, USA

**Keywords:** JNK, aging, lifespan, *Drosophila*

## Abstract

The evolutionarily conserved c-Jun N-terminal kinase (JNK) signaling pathway is a critical genetic determinant in the control of longevity. In response to extrinsic and intrinsic stresses, JNK signaling is activated to protect cells from stress damage and promote survival. In *Drosophila*, global JNK upregulation can delay aging and extend lifespan, whereas tissue/organ-specific manipulation of JNK signaling impacts lifespan in a context-dependent manner. In this review, focusing on several tissues/organs that are highly associated with age-related diseases—including metabolic organs (intestine and fat body), neurons, and muscles—we summarize the distinct effects of tissue/organ-specific JNK signaling on aging and lifespan. We also highlight recent progress in elucidating the molecular mechanisms underlying the tissue-specific effects of JNK activity. Together, these studies highlight an important and comprehensive role for JNK signaling in the regulation of longevity in *Drosophila*.

## 1. Introduction

Aging is characterized by the progressive decline of tissue/organ functions as well as cellular stress tolerance, which ultimately results in the death of an organism. Lifespan is therefore a robust measure of aging rate [1]. Genetic manipulations to prevent the age-associated deterioration of cellular functions and promote stress resistance/tolerance, can significantly extend lifespan [2]. Accordingly, signaling mechanisms that delay aging and/or increase stress protection are emerging as crucial regulators of longevity. Among them, the evolutionarily conserved c-Jun N-terminal kinase (JNK) signaling has emerged as a significant genetic determinant in the control of longevity [3]. JNK signaling is activated by various extrinsic and intrinsic stimuli—such as cytokines, growth factors, pathogens, toxins, drugs, oxidative stress, ultraviolet radiation, and DNA damage—triggering the reprogramming of the cells that is necessary to withstand these stresses and promoting organism survival.

*Drosophila melanogaster* has been used as a model organism to study aging and longevity for more than 100 years [4]. Flies with organism-wide JNK activity upregulation exhibit higher stress tolerance and longer lifespan [5,6,7]. Intriguingly, targeted genetic manipulation of JNK signaling in certain tissues/organs is sufficient to alter the lifespan of flies. However, the effects of tissue/organ-specific JNK signaling on lifespan and the underlying molecular mechanisms are versatile and context dependent.

In this review, we highlight the central role of JNK in the regulation of longevity in *Drosophila*, focusing specifically on the impact of JNK signaling in several tissues/organs that are highly associated with age-related diseases, such as metabolic organs (intestine and fat body), neurons, and muscles. We also illuminate recent progress in understanding the molecular mechanisms underlying the distinct, tissue-specific effects of JNK signaling.

## 2. *Drosophila* JNK Pathway Cascade

**Figure 1:**
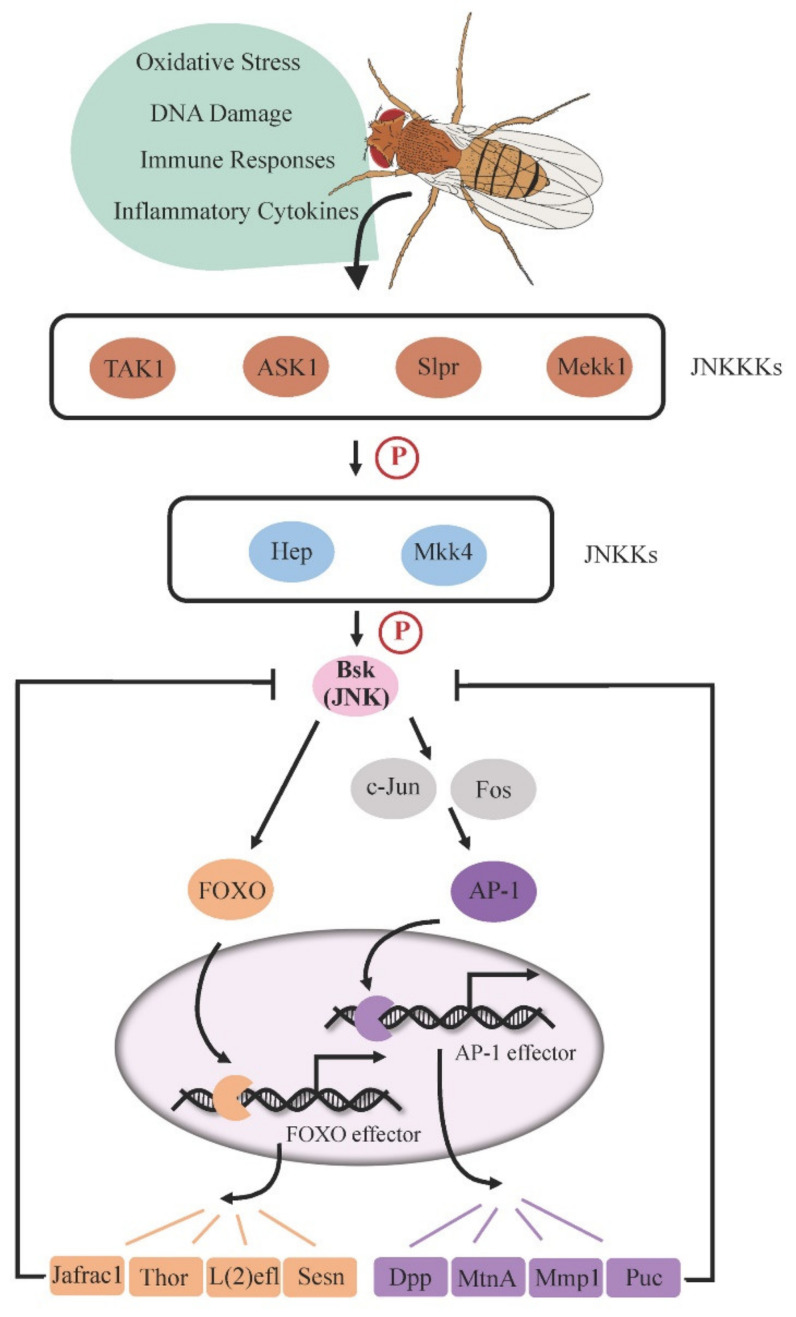
JNK pathway cascade in *Drosophila*. Stress-induced JNK signaling is initiated by JNK kinase kinases (JNKKKs), leading to the phosphorylation of JNK kinases (JNKKs) and subsequent Bsk (JNK) phosphorylation. Activated Bsk (JNK) drives downstream transcription factors AP-1 dimer or FOXO to trigger the expression of target genes. Several target proteins, such as Puc and Jafrac1, inhibit Bsk function, forming a negative feedback loop to regulate JNK signaling activity.

The JNK signaling pathway (Figure 1) is a kinase cascade composed of different MAPKs (mitogen-activated protein kinases) [8,9], which is primarily activated by stress factors, thus significantly impacting stress tolerance [10]. Whereas mammals express three JNK proteins (JNK1/2/3 or MAPK8/9/10), the *Drosophila* genome encodes a unique JNK protein by the gene *basket* (*bsk*), presenting a simple system to study this signaling.

Stress-stimulated JNK signaling is initially transduced by the JNK kinase kinases (JNKKKs), such as transforming growth factor beta activated kinase 1 (TAK1), apoptosis signal-regulating kinase 1 (ASK1), Slipper (Slpr), and MAPK kinase kinase 1 (Mekk1) [11,12,13,14]. JNKKKs then phosphorylate the JNK kinases (JNKKs), Hemipterous (Hep) or MAPK kinase 4 (Mkk4) [15], which subsequently phosphorylates downstream JNK (Bsk) on serine/threonine and tyrosine residues. In the canonical pathway, phosphorylated JNK activates the transcription factor AP-1, a heterodimer consisting of c-Jun and Fos (also known as Jra and Kay, respectively), triggering the expression of target genes required for a variety of cellular behaviors such as apoptosis and cell proliferation [8,9,10]. In particular, *puckered* (*puc*), one of the AP-1 target genes, encodes a phosphatase that dephosphorylates Bsk and thus functions as a negative feedback regulator to prevent cell oversensitivity to stress [16]. Alternatively, stress-responsive JNK can promote the nuclear localization of Forkhead Box O transcription factor (FOXO), which activates the expression of cytoprotective genes such as *thioredoxin peroxidase 1* (*Jafrac1*), *lethal (2) essential for life* [*l(2)efl*], and *thor* to advance stress defense [17,18]. It has been reported that overexpression of *Jafrac1* is able to suppress Bsk activity in neurons, indicating that Jafrac1 may also act as a negative feedback regulator of JNK in specific tissues [5].

In flies, the role of JNK signaling in the regulation of longevity was initially identified through organism-wide manipulation of JNK activity. Flies with mild activation of JNK signaling due to heterozygosity of *puc* loss-of-function alleles display a significantly longer lifespan than wild-type flies, and this phenotype is reduced in hemizygotes of *hep^1^*, a hypomorphic allele of *hep* [7], indicating that a functional JNK cascade extends lifespan. The downstream transcription factor FOXO is required for JNK-mediated lifespan extension, as the long-lived phenotype in *puc* mutants can be reverted to wild-type levels in the background of *foxo* loss-of-function allele heterozygotes [6]. Consistently, an extended lifespan was also observed in flies that ubiquitously overexpress FOXO target gene *l(2)efl* [6] or *hsp68* [7], suggesting that these FOXO-dependent stress-responsive genes could be the important effectors of JNK signaling in the control of longevity.

## 3. JNK in Aging Gut Shortens Lifespan

**Figure 2:**
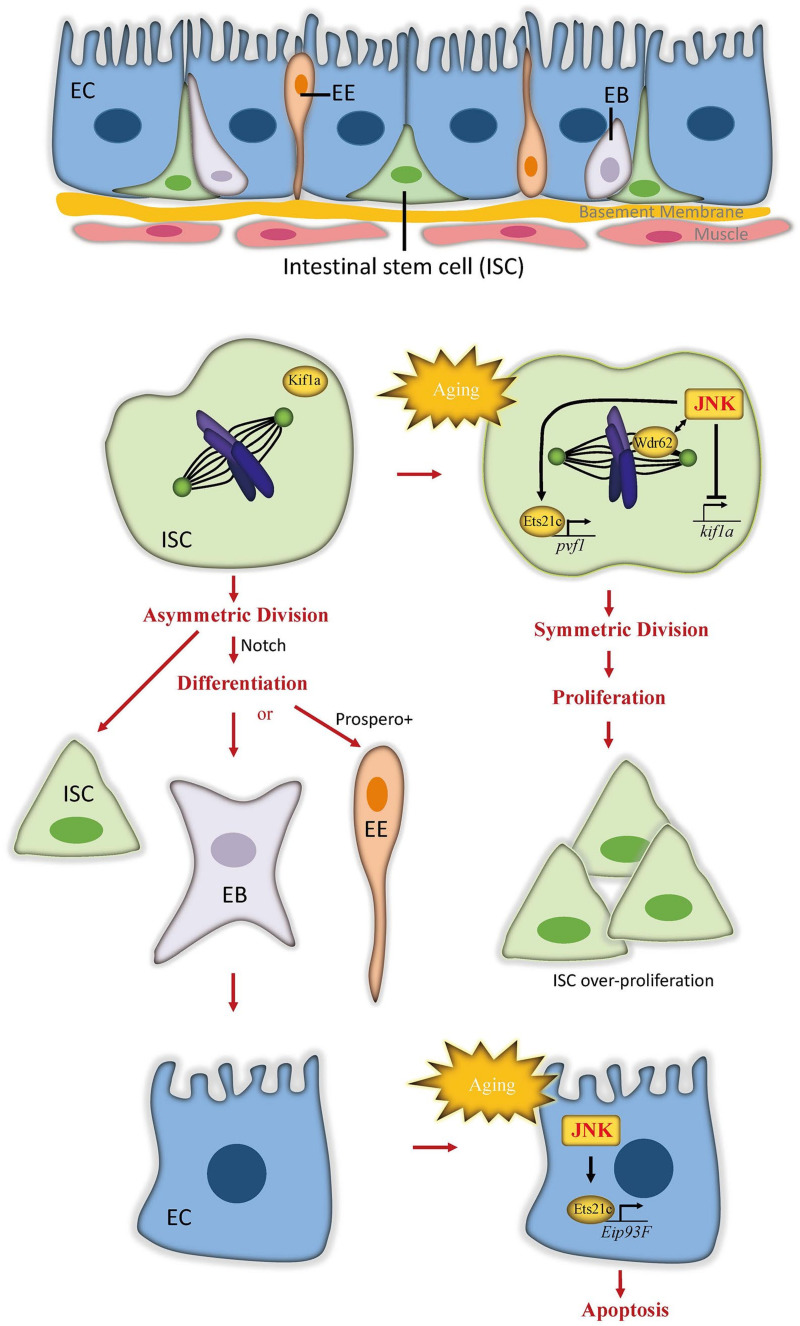
JNK signaling induces ISC proliferation and EC death in *Drosophila* aging intestine. In healthy gut, asymmetric division of intestinal stem cell (ISCs) regulated by Notch signaling generates both transient enteroblasts (EBs) and the enteroendocrine (EE) lineage-determined cells. EBs mature into large, polyploid, and polarized enterocytes (ECs). In aging gut, JNK signal is activated to induce the overproliferation of ISCs and apoptosis of ECs, which eventually result in dysplasia and disruption of intestinal integrity. In ISCs, JNK recruits and collaborates with Wdr62 to orient the spindle planar to the basement membrane, leading to symmetric cell division. At the transcriptional level, JNK signaling inhibits the expression of *kif1a*, a kinesin that promotes asymmetric division of ISCs but activates the transcription factor Ets21c to induce *pvf1* expression, which promotes ISC proliferation. In ECs, elevated JNK signaling activates Ets21c to trigger the expression of *Eip93F*, which subsequently initiates the apoptosis.

The intestine is the main organ to absorb nutrients from digested food to support body metabolism. It is also an important homeostatic organ acting as the first protective barrier of defense against ingested toxins and pathogens. The adult *Drosophila* midgut, which is functionally equivalent to the mammalian intestine, has been established as a model system to study homeostasis and aging because of its simple structure and well-defined cell lineages [19]. The intestinal epithelium is composed of a monolayer of nutrient-absorbing enterocytes (ECs), with basally embedded intestinal stem cells (ISCs) and apically located secretory enteroendocrines (EEs) (Figure 2). ISCs, marked by expression of the Notch ligand Delta, self-renew in symmetrical division mode [20]. Through asymmetrical division, ISCs generate either transient enteroblasts (EBs), which possess high levels of Notch signaling activity and terminally differentiate into ECs, or differentiated EEs when the neuronal transcription factor Prospero is highly expressed [21,22,23]. To replace damaged cells and preserve gut integrity, the midgut epithelium maintains a high turnover rate of 1–2 weeks [24,25]. Defective renewal and integrity of the intestine are considered the main cause of intestinal function decline and lifespan shortening [26].

### 3.1. The Role of JNK in the Aging Gut

As a stress-responsive signal, JNK signaling activity is required for stress protection in intestinal cells, which induces ISC proliferation and EC apoptosis to promote intestinal epithelial turnover and thus protect adult flies from stress damage [25,27,28,29]. Aging shifts ISC division progressively toward the symmetric mode, creating a massive ISC population in aged gut. Due to the loss of intestinal homeostasis, the old gut is also characterized by the accumulation of misdifferentiated cells. Together, these changes cause epithelial dysplasia and a breakdown of epithelia integrity [27,30]. Strikingly, JNK signaling activity in the gut chronically increases with age [27], and appears to contribute to, rather than ameliorate, the age-related deterioration of the intestinal epithelium. In *hep^1^* mutants or in flies with reduced JNK activity specifically in ISCs and EB cells, the morphological and structural abnormalities of the old gut are significantly reduced [27]. Conversely, JNK activation in ISCs and EBs is sufficient to induce age-associated ISC population enlargement. Elevated JNK signaling in the old gut is also speculated to disturb the asymmetric segregation of Delta protein [27], resulting in the misregulated differentiation of ISCs. Differently, excessive JNK signaling activities in ECs causes the loss of ECs and compensatory ISC proliferation, which is also associated with a short lifespan [31,32,33]. Taken together, these results suggest that the age-induced JNK activity in the old gut mainly play a negative role in the longevity of flies.

Age-associated ISC overproliferation and misdifferentiation increase the risk of tumorigenesis in the gut, which dramatically shortens the lifespan of flies. JNK signaling plays both anti- and pro-tumorigenic roles during tumor progression (recently reviewed in [34]). It inhibits tumorigenesis by inducing apoptotic death in targeted cells. However, if JNK-induced apoptosis is suppressed, JNK signaling in tumor cells promotes both autonomous and non-autonomous cell proliferation as well as tumor cell invasiveness, accelerating tumor malignancy. In the intestinal tumor models, JNK signaling primarily exerts a pro-tumorigenic effect. For example, in a *Drosophila* intestinal tumor model caused by the loss of bone morphogenetic protein (BMP) signaling, aberrant activation of JNK signaling was observed in tumors and linked to intestinal barrier dysfunction [35]. Inhibiting JNK signaling restores barrier function of the intestinal epithelium. Similar effects were also reported in a mouse intestinal tumor model caused by elevated β-catenin signaling. Inactivation of *c-jun* reduces the tumor number and size and prolongs lifespan [36].

### 3.2. Distinct Molecular Mechanisms of JNK Function in Different Intestinal Cells

The molecular mechanisms underlying the effects of JNK signaling in the aging gut have been found to differ among intestinal cell lineages in *Drosophila*. In ISCs, increased JNK signaling activity in the aging gut promotes symmetric ISC division by regulating spindle orientation [20]. Planar spindle orientation contributes to the symmetric division outcome, and this orientation predominates in mitotic ISCs in older flies. Blocking JNK activity via expressing a dominant-negative form of JNK (*bsk^DN^*) can prevent age-induced planar spindle orientations in dividing ISCs. Conversely, excessive activation of JNK in ISCs by overexpressing *hep* or by knocking down *puc* is sufficient to induce planar spindle orientation. JNK signaling biases planar spindle orientation and symmetric division of ISCs through collaboration with WD40-repeat protein (Wdr62) [20], a spindle-associated protein [37,38]. In addition, JNK inhibits the formation of oblique spindles used in asymmetric division by promoting the release of Mud protein from the cell cortex [20], where it is required to orient oblique spindles. At the transcription level, JNK represses the expression of *kif1a*, a kinesin reported to inhibit symmetric divisions in brain neural stem cells [39]. The overexpression of *kif1a* prevents JNK-induced planar spindle orientation but cannot affect spindle orientation in conditions without JNK activation [20], indicating that the repression of *kif1a* serves as a downstream step of JNK activation in the regulation of spindle orientation and ISC division. Consistent with the finding that moderate repression of JNK activity in intestinal progenitor cells extends lifespan [30], suppressing planar spindle orientation significantly increases lifespan in flies [20].

Unlike ISCs, ECs respond to increased JNK activation by initiating apoptosis. The transcription factor Ets21c was identified as a downstream effector of JNK function in ECs [28]. Ets21c, the ortholog of human proto-oncogenes FLI1 and ERG, belongs to the transcription factor family of E-twenty-six (ETS) and is transcriptionally expressed in response to stress and aging [40,41,42,43]. Interestingly, Ets21c also mediates JNK-induced ISC proliferation. To dissociate the proliferative and proapoptotic functions of JNK signaling, Mundorf et al. demonstrated that Ets21c activates distinct sets of target genes in ISCs and ECs to promote cell proliferation or cell death [28]. The JNK-Ets21c signal in ISCs activates downstream PDGF- and VEGF-related factor 1 (Pvf1), which then induces ISC proliferation. In ECs, however, JNK-activated Ets21c triggers apoptosis through its target gene *Ecdysone-induced protein 93F* (*Eip93F*) [27], which encodes a transcription factor known to play a role in programmed cell death in the midgut and fat body during fly metamorphosis [44,45]. The JNK-Ets21c signal in ECs also activates the expression of the cytokine gene *upd3* and the growth factor gene *pvf1*, which promote ISC compensatory proliferation through the JAK/STAT pathway or PDGF/VEGF receptor (Pvr) signaling, respectively. As with reduced JNK signaling in the gut, loss of *ets21c* prolongs lifespan but renders flies sensitive to stress. Overall, JNK signaling employs distinct molecular mechanisms to modulate ISC proliferation and EC apoptosis, and the age-related upregulation of JNK signaling progressively disturbs the regenerative process and the integrity of the intestinal epithelium through these mechanisms.

Aging-related upregulation of JNK signaling in the gut may be attributable to rising concentrations of reactive oxygen species (ROS). The presence of ROS induces JNK signaling in many tissues [46,47,48]. Mitochondria, the primary producers of cellular ROS, are particularly susceptible to damage and malfunction during aging across species [49,50,51]. Recently, Dai et al. [33] showed that deletion of *caliban* (*clbn*), a gene that encodes a mitochondria outer membrane protein, induced mitochondrial damage as well as typical hallmarks of aging in the intestinal epithelium: EC damage, ISC overproliferation, disruption in the intestinal barrier, and shortened lifespan. In addition, *clbn* mutants exhibited elevated ROS levels and elevated JNK signaling in the gut. Blocking JNK activity in *clbn* mutant ECs largely rescued the intestinal epithelial abnormalities and longevity phenotypes but not the defective mitochondrial morphology, indicating that mitochondrial dysfunction and ROS overproduction act upstream of JNK-induced gut defects.

## 4. JNK in Brain/Neurons Extends Lifespan

Aging is considered a primary risk factor for most neurodegenerative diseases [52], including Alzheimer’s and Parkinson’s diseases, which severely reduce the quality of life and longevity. Interestingly, increasing JNK activity specifically in brain neurons is sufficient to promote stress tolerance and extend the lifespan of the whole organism [6,7], suggesting that neuronal JNK is critical for the JNK-mediated extension of the *Drosophila* lifespan. Here, we summarize evidence that neuronal JNK exerts its effects through multiple intersecting mechanisms: regulation of neuronal proteostasis, antagonism of insulin signaling, and protection from oxidative stress.

Optimal proteostasis is important for neuronal functions. Dysfunction in proteostasis frequently underlies neurodegenerative disease pathologies [53]. In these cases, abnormal proteins accumulate in brain cells and cannot be effectively cleared [52]. Similar complications arise as a result of aging—the protein turnover rate is globally reduced in older brains [54], indicating an age-related breakdown in proteostasis. Interestingly, this age-induced reduction in the protein turnover rate was not observed in old heads carrying a loss-of-function mutation in *puc* [54], a downstream negative regulator of JNK signaling (Figure 1). *puc* mutants exhibit elevated JNK activity and live significantly longer than wild-type controls. Further supporting a positive role of JNK signaling for proteostasis and longevity, pan-neuronal overexpression of the JNK/FOXO target gene *l(2)efl*, encoding a member of the heat-shock protein family that assists in protein folding and prevents accumulation of misfolded proteins, is sufficient to extend lifespan.

Autophagy, a conserved lysosomal degradation pathway for damaged protein, lipids, and organelles [55], plays a critical role in the maintenance of cellular proteostasis. Mutations in the autophagy-related (*atg*) gene result in the accumulation of protein aggregates in neurons and a shortened lifespan [56], whereas the enhanced expression of *atg* gene in the brain is sufficient to extend the average adult lifespan [57]. JNK signaling induces the expression of *atg* genes [58] and promotes autophagy [59,60], highlighting an involvement of autophagy in the regulation of JNK signaling on lifespan. Therefore, activating JNK in the aging brain could more effectively maintain proteostasis and thus extend the lifespan.

The association between neuronal JNK and longevity has been well elucidated in a small cluster of neuroendocrine cells in the brain, known as insulin-producing cells (IPCs). These cells secret insulin-like peptides (ILPs) [61,62], which function similarly to human insulin, to stimulate the insulin/IGF signal (IIS) pathway, a well-known signal transduction system controlling metabolism. The *Drosophila* genome contains seven *dilp* genes encoding ILPs, each expressed in a stage- and tissue-specific pattern, such as Dilp2 in IPCs and Dilp6 in fat bodies. JNK signaling is active in these IPCs, activating FOXO and restricting the expression of ILP genes to systemically antagonize IIS in peripheral tissues [6]. When nutrients are abundant, IIS promotes tissue growth and energy storage but shortens lifespan [6,63]. Conversely, JNK-mediated repression of ILP genes in IPCs has no effect on organism growth but significantly extends lifespan [6]. 

**Figure 3:**
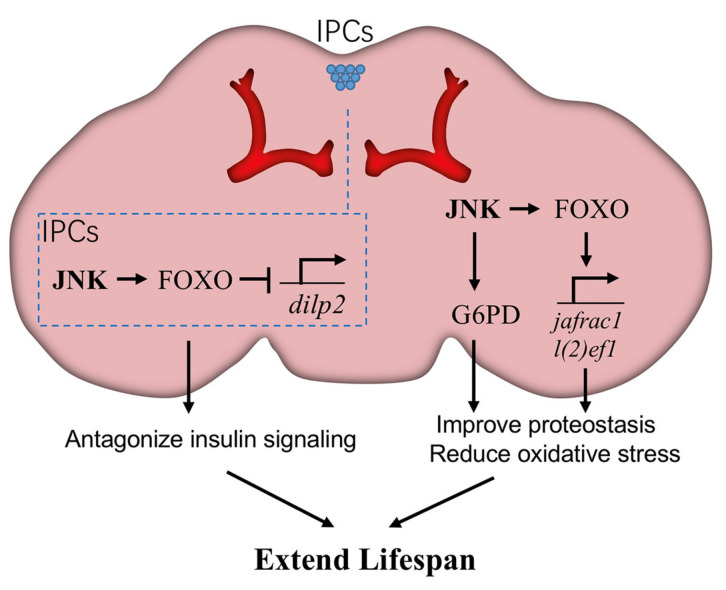
JNK signaling in neurons promotes *Drosophila* lifespan. JNK signaling in neurons triggers the expression of glucose-6-phosphate dehydrogenase (G6PD) and then increases NADPH production, which is beneficial to reduce oxidative stress. Meanwhile, JNK activates the downstream transcription factor FOXO to induce the expression of cytoprotective genes, such as *l(2)efl* and *Jafrac1*, against age-related damage. Particularly in brain insulin-producing cells (IPCs), JNK-FOXO inhibits the expression of *Drosophila ilp2* (*dilp2*), thereby blocking insulin signaling in peripheral tissues. Each of these effects is beneficial for longevity.

The brain consumes a large amount of oxygen for proper function and consequently produces high levels of ROS, thus it is highly susceptible to oxidative damage [64]. JNK signaling in neurons enhances stress resistance and extends lifespan through several downstream effectors (Figure 3). For example, JNK signaling increases expression of *Jafrac1*, a peroxiredoxin that detoxifies peroxides. *Jafrac1* is expressed in the adult brain and upregulated by paraquat stress [5]. Neuronal knockdown of *Jafrac1* shortens, while overexpression of *Jafrac1* extends, the lifespan of flies [5].

Recently, Wang et al. explored another mechanism through which JNK signaling reduces oxidative stress in neurons and enhances longevity [54]. In *puc* mutants, JNK signaling in brains induces the expression of *G6PD* (glucose-6-phosphate dehydrogenase). In turn, G6PD oxidizes glucose-6-phosphate, an intermediate of glycolysis, by transferring hydrides to NADP+, forming NADPH. The increase of cytosolic NADPH defends against oxidative stress by reducing the concentration of oxidized glutathione. Intriguingly, overexpression of *G6PD* specifically in neurons is sufficient to mimic the proteostatic and longevity effects of JNK gain-of-function conditions, suggesting an important contribution of G6PD in neuronal JNK-induced lifespan extension. Consistent with this hypothesis, *G6PD* overexpression was also shown to improve lifespan in a mouse model [65], although the genetic interaction between JNK and G6PD remains unclear.

In addition, increasing evidence suggests a positive role of sleep in resistance to oxidative stress [66,67], which is required for a healthy lifespan. Sleep homeostasis mechanism is thought to be neuronally based [68,69,70], and JNK signal appears to regulate this process—knocking down JNK in neurons leads to fragmented sleep patterns and a shorter lifespan [71]. Taken together, existing evidence supports a model in which neuronal JNK signaling prolongs lifespan through multiple cellular mechanisms.

## 5. JNK in Fat Body Delays Aging

The *Drosophila* fat body is analogous to mammalian white adipose tissue and liver, performing metabolic and immune functions [72,73], both of which are closely linked to the regulation of fly longevity. 

**Figure 4:**
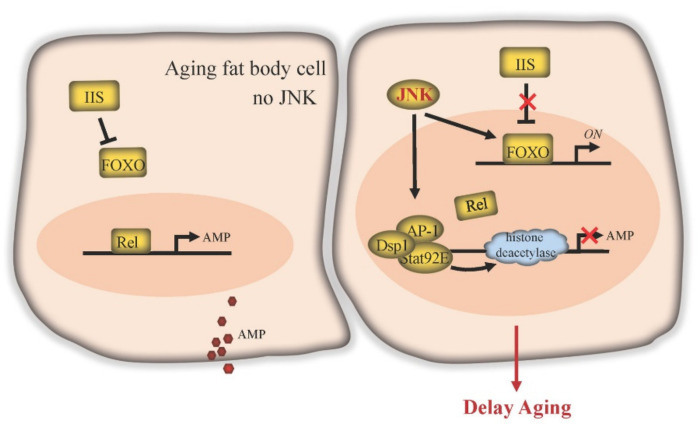
JNK signaling delays aging in *Drosophila* fat body. IIS inhibits FOXO function. The transcription factor Relish (Rel) of the IMD pathway can be activated in old fat body cells to activate the expression of downstream target antimicrobial peptide (AMP) genes. Elevated expression of AMP can induce apoptosis, elicit depolarization of mitochondria and significantly shorten lifespan. JNK signaling blocks the effects of IIS, enabling expression of FOXO target genes. JNK activates the transcription factor AP-1, which forms a repressosome complex in the nucleus together with Stat92E and Dsp1. This complex competitively blocks Rel binding to immune effector gene promoters and recruits ambient histone deacetylase to inhibit AMP gene transcription.

In fat body cells, JNK signaling facilitates lifespan control by counteracting IIS activity (Figure 4). The important role of fat tissue IIS in the regulation of lifespan has been consistently demonstrated in both *Drosophila* and mammals. In mice, deletion of the insulin receptor in white adipose tissue results in a lean, long-lived phenotype [74]. In *Drosophila*, reduction of IIS pathway activity in fat bodies also extends lifespan [75,76]. The IIS pathway is activated by insulin or insulin-like peptides (ILPs) and regulates gene expression by causing cytoplasmic retention of FOXO and repression of FOXO target gene expression. Consistent with the negative role of IIS in lifespan, overexpression of *foxo* in fat bodies is sufficient to extend lifespan [76,77]. JNK signaling antagonizes IIS, allowing the nuclear localization of FOXO cell-autonomously and the expression of its targets, including the IIS ligand gene *dilp6*. Expression of *dilp6* then systemically represses *dilp2* production in brain neuroendocrine cells, ultimately reducing the IIS activity in the peripheral tissues [6,75,76].

The fat body is also a well-known immune tissue in *Drosophila*. It defends against bacteria and viruses by producing a large number of antimicrobial peptides (AMPs). Two immune pathways, the IMD (immune deficiency) pathway and the Toll pathway, are responsible for AMP gene expression in fat bodies, through the downstream NF-κB transcription factors Relish or Dif/Dorsal, respectively [78]. Under conditions of bacterial infection, the immune response of fat bodies and the production of AMP genes are required for fly survival. *Relish* loss-of-function homozygotes lose the ability against infection and quickly die [79]. However, in the absence of bacterial infection, AMPs are likely harmful for flies, as overexpression of *Relish* or the individual AMP genes globally or specifically in fat bodies induces apoptosis, elicits depolarization of the mitochondria, and significantly shortens lifespan [80]. Strikingly, it has been found that the expression of *Relish* and AMP genes chronically increases during aging. Eliminating Relish activity prevents age-dependent upregulation of AMPs and extends lifespan [80]. Similarly, fat body-specific downregulation of AMP expression also enhances lifespan [81], suggesting that IMD-induced AMP production could play a role in the aging progress.

JNK signaling in fat body cells is a potent candidate to repress AMP expression. After bacterial infection, *Relish* loss-of-function homozygotes in the *Jra* (c-Jun in *Drosophila*) heterozygous mutant background exhibit higher levels of AMP transcripts and a higher survival rate within 5 days [82]. In addition, in cultured cells, JNK pathway transcription factor AP-1 forms a repressosome complex together with Stat92E. This complex then blocks the expression of immune effector genes (such as AMPs) by competitively blocking Relish binding at the promoter and by recruiting ambient histone deacetylase [82]. Although further in vivo data is required, this repressing machinery suggests that JNK signaling may play a cytoprotective role in fat body cells by inhibiting the IMD/Relish pathway to limit the bacterial-induced excessive immune response, and restrict the age-dependent increase in AMP levels, thus delaying aging and extending lifespan. 

## 6. JNK Preserves Youthful Muscle Function

Muscle function decline is a common degenerative event during aging and is associated with many age-related diseases [83]. In *Drosophila*, muscle function gradually decreases with age, resulting in impaired climbing and flight abilities [84]. Many age-associated changes are particularly pronounced in fly muscle cells [83], including age-related apoptosis, accumulation of damaged proteins, and increased mitochondria damage. 

**Figure 5:**
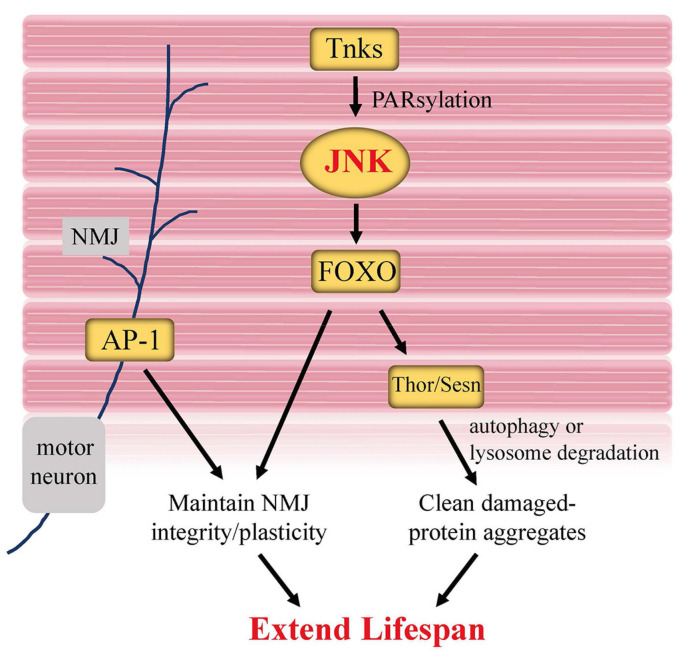
JNK signaling in muscle modulates *Drosophila* lifespan. Tankyrase (Tnks), a member of the poly(ADP-ribose) polymerase (PARP) superfamily, mediates the K63-linked polyubiquitination of Bsk (JNK) via PARsylation, promoting JNK activity. Increased JNK activity caused by Tnks in muscle cells is considered to be able to improve stress tolerance and extend lifespan. In addition, AP-1 activity in motor neurons is able to maintain NMJ integrity and plasticity, thus keeping muscle and NMJ youthfulness.

As in other tissues, genetic manipulation affecting the JNK pathway in muscle can influence aging and longevity in flies. Flies with reduced JNK in muscle exhibit impaired climbing ability, increased sensitivity to paraquat and starvation [85]. Interestingly, Li et al. recently discovered a muscle-specific posttranslational modification of JNK protein during lifespan regulation [85]. Tankyrase (Tnks), which belongs to the poly(ADP-ribose) polymerase (PARP) superfamily, functions to transfer ADP-ribose from NAD+ onto substrate proteins [86]. This posttranslational modification is referred to as PARsylation. Tnks catalyzes the PARsylation of the K63-linked polyubiquitination of Bsk protein to promote JNK kinase activity (Figure 5). Adult *Tnks* mutants survive significantly shorter and show apparent decline in climbing ability as well as impaired stress tolerance. Tissue-specific analysis suggests that Tnks principally mediates lifespan via its activity in the muscle, rather than in fat bodies, intestine or neurons. Given that knockdown of *Tnks* strongly decreases the activity of Bsk [85,87], Tnks-mediated PARsylation of JNK in muscle positively regulates lifespan.

Aging muscles progressively accumulate damaged proteins due to defects in protein homeostasis [84], which is highly associated with impaired muscle functions. Expression of JNK/FOXO and their targets, Thor and Sesn, is sufficient to delay the thoracic muscle weakness and extend longevity by promoting the activity of the autophagy/lysosome degradation system to remove damaged protein aggregates [84,88]. 

Accurate and fast muscle function requires crosstalk between muscles and motor neurons. The neuromuscular junction (NMJ) serves as the synaptic interface between the branched terminals of motor neurons and the muscle fibers. Fos and Jun, two components of AP-1, are abundantly expressed in motor neurons. Induction or inhibition of AP-1 in motor neurons is necessary and sufficient to modulate NMJ size and strength [89,90]. Most recently, Birnbaum et al. [91] found that young flies carrying a null mutation in *foxo* gene exhibited “old” NMJ morphology in the abdominal ventral longitudinal muscles, including larger synaptic bouton areas and shorter terminal branches. Aging neurons undergo cytoskeletal changes in order to maintain plasticity and synaptic contact to muscles. Upregulation of late endocytic vesicles indicates a disruption of motor neuron homeostasis. Birnbaum et al. found that motor neuron-specific overexpression of *foxo* delays these age-related changes to NMJ morphology, suggesting that FOXO activity maintains morphological and synaptic plasticity at the aging NMJ. Together, Foxo and AP-1 are both beneficial for maintaining “young” muscle and NMJ.

## 7. Mammalian JNK in Aging and Age-Related Disease Models

JNK signaling is evolutionarily conserved in eukaryotes. Its conserved functions of inducing apoptosis and/or cytoprotective responses have been demonstrated in both *Drosophila* and mammalian systems. However, the role of JNK signaling in normal physiological aging processes and in longevity remains elusive in mammals. The long lifespan of mammalian models may pose a major obstacle to such studies.

As an alternative, the age-related disease models have been repeatedly used to understand the function of JNK signaling. For instance, in mouse models of Alzheimer’s disease (AD), the most frequent neurodegenerative disorder, JNK3 phosphorylation levels correlate with the progression of AD, thus, it serves as a biomarker for AD. Mechanically, JNK3 phosphorylates the amyloid precursor protein (APP), causing amyloidogenic proteolytic processing and Aβ production in the brain [92]. In mouse models of Parkinson’s disease (PD), another common neurodegenerative disease, JNK is involved in the activation of neuronal cell apoptosis through suppressing the function of antiapoptotic factor Bcl-2 [92]. Thus, inhibition of the JNK signaling is considered to be a protective strategy against the neurodegeneration in AD and PD, and the JNK inhibitor has been proposed as the therapeutic candidate for these diseases [92].

JNK signaling has also been examined in mouse models of disease outside of the nervous system. In a mouse model of liver injury caused by acetaminophen overdose, JNK promotes hepatocyte death. Upregulation of JNK activity by knocking out MAPK phosphatase 1 (Mkp-1), a negative regulator of JNK, exacerbated hepatic injury and shortened survival time [93]. Similarly, in a high fat diet (HFD)-induced liver injury model, knocking out JNK-interacting protein 3 (JIP3), a scaffold protein that can bind to JNK1/3, protected mice against HFD-induced liver injury in part through decreasing hepatic p-JNK level [94]. Together, these studies suggested a negative role of JNK activity for liver health. In contrast, in an osteoarthritic (OA) mouse model, deletion of JNK1/2 enhances the severity of age-related OA [95], suggesting a protective role of JNK in musculoskeletal tissues during aging. Collectively, studies in mice suggest that mammalian JNK exerts widespread and context-dependent effects in models of aging and diseases.

## 8. Conclusions and Perspective

JNK signaling is a significant genetic determinant of aging and longevity in *Drosophila*. Ubiquitous elevation of JNK signaling activity in the whole organism can delay aging and extend lifespan. Moreover, manipulating JNK signaling in a specific tissue/organ, such as intestine or neurons, is sufficient to alter fly lifespan; however, these effects may be positive or negative depending on the context. For example, lifespan is extended when JNK is activated in the brain, whereas moderate activation of JNK in midgut ISCs and EBs leads to a significant shortening of lifespan. Therefore, efforts to dissect the mechanism underlying JNK function in the determination of longevity should be conducted in a tissue/organ-specific manner.

The classic function of JNK signaling is to trigger apoptosis for the clearance of abnormal or needless cells [96,97,98,99]. However, increased apoptosis during aging, as observed in gut, muscle, fat, and select subsets of neurons [100,101], results in the destruction and degeneration of these tissues. Accordingly, reducing apoptosis in these tissues might be a feasible strategy to delay aging. Indeed, it has been recently reported that apoptosis inhibition in *Drosophila* mitigates the effects of aging and prolongs lifespan [102].

In addition to its proapoptotic function, JNK signaling functions as a stress-activated signal to induce cytoprotective cellular responses, including autophagy, DNA repair, and antioxidant enzyme induction. In aging tissues/organs where increased apoptosis gradually accelerates cell loss, JNK activity shifts toward a cytoprotective role rather than a proapoptotic role, promoting cell repair and restricting apoptosis to improve tissue/organ homeostasis. In the aging fat bodies and neurons, JNK antagonizes the insulin signaling and activates FOXO, which in turn switches on the expression of a subset of cytoprotective genes to protect cells from various stress challenges. In addition, JNK signaling in muscles and neurons improve proteostasis to delay aging. In contrast, in tissues with a high rate of cell turnover, such as the intestinal epithelium, age-induced JNK activity persists to trigger cell death and subsequent regenerative cell proliferation, mainly contributing to a decline in homeostasis. Outside of these limited exceptions, however, increasing JNK signaling in aging tissues/organs most often protects cells from death and extends lifespan.

To understand the different outcomes of JNK signaling during aging, that is, its proapoptotic effects in young organisms and its cytoprotective effects in aging organisms, the upstream signaling is an important consideration. Numerous studies have revealed that JNK signaling during development and young adulthood can be activated by developmental cues as well as extrinsic or intrinsic stressors [103,104,105]. In contrast, the upstream signals that activate the JNK pathway in aging flies are less clear. Several recent studies have implicated age-associated ROS production from mitochondria in the induction of JNK signaling. Age-induced mitochondria damage enhances the production of ROS, which functions as an upstream signal molecule in the activation of the JNK pathway [33,60,102]. Generally, proapoptotic responses serve as a mechanism to clear cells following severe cellular damage, whereas cytoprotective cellular responses to JNK signaling trigger mechanisms that help to repair cells upon a minor damage. Unlike the acute developmental signals and stressors encountered by young cells, age-associated mitochondrial dysfunction and ROS production provide weaker chronic and cumulative signals to aging cells. These weaker signals may induce cytoprotective reactions but might not be strong enough to trigger apoptosis.

In the past decades, researchers have achieved significant progress in unraveling the molecular mechanisms and consequences of JNK signaling on aging and lifespan in *Drosophila*. Studies in *C. elegans* have also revealed the significant contributions of JNK signaling in longevity and survival following bacterial infections [106,107]. The mammalian JNK-MAPK pathway plays a comprehensive role in the age-related disease models, although an understanding of its function in determining lifespan remains elusive. Knowledge obtained from these ongoing studies in model organisms will likely provide the theoretical bases for future methods to improve aging health and extend lifespan in humans.
